# Pellucid Marginal Degeneration: A Comprehensive Review of Pathophysiology, Diagnosis, and Management Strategies

**DOI:** 10.3390/jcm14155178

**Published:** 2025-07-22

**Authors:** Michael Tsatsos, Konstantina Koulotsiou, Ioannis Giachos, Ioannis Tsinopoulos, Nikolaos Ziakas

**Affiliations:** 1 2nd Ophthalmology Department, Papageorgiou Hospital, Aristotle University of Thessaloniki, 541 24 Thessaloniki, Greece; ioannis.tsinopoulos@gmail.com (I.T.); nikolasziakas@gmail.com (N.Z.); 2 Ophthalmology Postgraduate Program, Aristotle University of Thessaloniki, 541 24 Thessaloniki, Greece; konkoulotsiou@yahoo.gr (K.K.); giaxosgiannis@hotmail.com (I.G.)

**Keywords:** pellucid marginal degeneration, corneal ectasia, corneal graft, corneal wedge excision, CAIRS

## Abstract

**Purpose:** Pellucid Marginal Degeneration (PMD) is a rare ectatic corneal disorder characterized by inferior peripheral thinning and significant irregular astigmatism. Despite its clinical similarities to keratoconus, PMD presents unique diagnostic and therapeutic challenges. This review aims to provide a comprehensive update on the pathophysiology, clinical features, diagnostic approaches, and management strategies for PMD, emphasizing the latest advancements in treatment options. **Methods:** A systematic literature search was performed in MEDLINE (via PubMed), Google Scholar, and Scopus up to February 2025 using the terms: “pellucid marginal degeneration,” “PMD,” “ectatic corneal disorders,” “keratoplasty in PMD,” “corneal cross-linking in PMD,” “ICRS in PMD,” “toric IOL PMD” and their Boolean combinations (AND/OR). The search was restricted to English-language studies involving human subjects, including case reports, case series, retrospective studies, clinical trials, and systematic reviews. A total of 76 studies met the inclusion criteria addressing treatment outcomes in PMD. **Results:** PMD is characterized by a crescent-shaped band of inferior corneal thinning, leading to high irregular astigmatism and reduced visual acuity. Diagnosis relies on advanced imaging techniques such as Scheimpflug-based corneal tomography, which reveals the characteristic “crab-claw” pattern. Conservative management includes rigid gas-permeable (RGP) lenses and scleral lenses, which provide effective visual rehabilitation in mild to moderate cases. Surgical options, such as CXL, ICRS, and toric IOLs, are reserved for advanced cases, with varying degrees of success. Newer techniques such as CAIRS, employing donor tissue instead of synthetic rings, show promising outcomes in corneal remodeling with potentially improved biocompatibility. Penetrating keratoplasty (PK) and deep anterior lamellar keratoplasty (DALK) remain definitive treatments for severe PMD, though they are associated with significant risks, including graft rejection and postoperative astigmatism. **Conclusions:** PMD is a complex and progressive corneal disorder that requires a tailored approach to management. Early diagnosis and intervention are critical to optimizing visual outcomes. While conservative measures are effective in mild cases, surgical interventions offer promising results for advanced disease. Further research is needed to refine treatment protocols and improve long-term outcomes for patients with PMD.

## 1. Introduction

Pellucid marginal corneal degeneration (PMD) is a rare corneal ectatic disorder characterized by bilateral peripheral thinning of the cornea. This thinning typically presents as a crescent-shaped band, most frequently observed in the inferior quadrant, though other regions may also be affected [1,2]. The ectatic zone tends to be located more centrally, while the area of maximal thinning is usually situated 1–2 mm from the limbus [3,4].

PMD predominantly affects males in their second to fifth decades of life, often leading to reduced visual acuity due to high irregular against-the-rule astigmatism. Similar to keratoconus, PMD often demonstrates asymmetry in the extent and severity of corneal thinning between the two eyes. However, this asymmetry is less frequently emphasized in the literature, likely due to the rarity and variable clinical presentation of PMD [5]. Notably, PMD is far less common than keratoconus (KC), the most prevalent corneal ectasia [6,7]. The exact aetiology of PMD remains unclear, with several hypotheses proposed but no definitive consensus [8]. Furthermore, it is still debated whether PMD, KC, and keratoglobus represent distinct entities or phenotypic variations of the same spectrum [9,10,11]. Some researchers [2,11,12,13,14] have proposed that PMD may be a peripheral variant of KC, a notion supported by reports of concurrent PMD and KC in the same eye. For instance, Kayazawa et al. [15] documented KC features in 17 out of 20 PMD patients.

Despite available treatment options, the literature on PMD remains scarce, primarily consisting of case reports and small case series, with few large-scale studies [2]. This gap is further complicated by the frequent inclusion of mixed PMD–KC cohorts in research, making it difficult to draw conclusions specific to PMD alone.

## 2. Pathophysiology and Epidemiology

Similar to keratoconus, the pathophysiology of PMD remains incompletely understood. Histopathological studies have revealed structural abnormalities, including an absent or irregular Bowman’s membrane with focal breaks and occasional Descemet’s membrane folds [4]. Electron microscopy further demonstrates irregularly spaced collagen fibres, with an increased intracollagen space periodicity of 100–110 nm, compared to the normal range of 60–64 nm in healthy corneas [8]. These findings suggest an inherent biomechanical weakness in the corneal stroma, which may contribute to the disease’s progression.

Historically, PMD and other ectatic disorders were classified as non-inflammatory conditions [16]. However, emerging evidence challenges this view. For instance, McKay et al. [17] identified a link between endocrine dysfunction, pro-inflammatory metabolites, and the presence of KC. Additionally, studies have highlighted a potential association between sex hormones and KC, raising questions about similar mechanisms in PMD [18].

PMD exhibits a gender predilection (being much more prevalent in males with a 3:1 male to female ratio), though no clear ethnic predisposition has been established [8,9,19]. Given the shared pathophysiological features between PMD and other ectatic disorders, some researchers propose that these conditions may represent phenotypic variations of a single disease spectrum. Systemic factors, such as obesity and obstructive sleep apnea, have also been implicated in PMD [3]. Furthermore, ocular comorbidities, including atopic eye disease, are hypothesized to play a role in its development or progression [4,20].

Methods: A systematic literature search was performed in MEDLINE (via PubMed), Google Scholar, and Scopus up to February 2025 using the terms: “pellucid marginal degeneration,” “PMD,” “ectatic corneal disorders,” “keratoplasty in PMD,” “corneal cross-linking in PMD,” “ICRS in PMD,” “toric IOL PMD” and their Boolean combinations (AND/OR). The search was restricted to English-language studies involving human subjects, including case reports, case series, retrospective studies, clinical trials, and systematic reviews. Of the 237 initially identified articles, 98 were excluded after title/abstract screening for irrelevance or duplication, and 63 were excluded after full-text review due to insufficient PMD-specific data (e.g., mixed keratoconus/PMD cohorts without subgroup analysis). A total of 76 studies met the inclusion criteria, addressing diagnosis, pathophysiology, clinical features, and treatment outcomes in PMD. Given the heterogeneity and descriptive nature of the included studies, a narrative review framework was adopted, and no formal risk-of-bias assessment was conducted. Both conservative and surgical management options were evaluated, including contact lenses, collagen cross-linking (CXL), intrastromal corneal ring segments (ICRS), toric intraocular lenses (IOLs), and various keratoplasty techniques.

## 3. Clinical Signs

**Figure 1:**
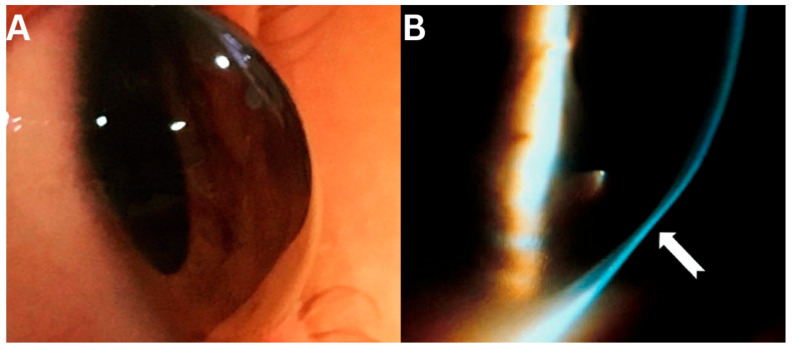
Slit-lamp image collage of PMD. Picture (**A**) shows inferior thinning with a beer-belly appearance while picture (**B**) shows the ectatic zone extending in a crescent-shaped pattern from the 4 to 8 o’clock positions. Arrow pointing the area of maximal thinning typically located 1–2 mm from the limbus.

**Figure 2:**
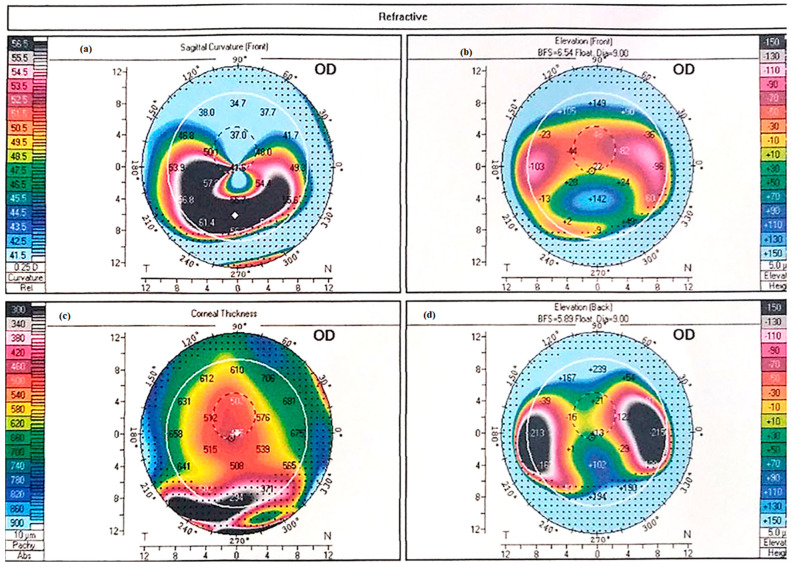
Tomographic image of Axial Sagittal Curvature map showing the crab claw appearance of PMD on (**a**) sagittal curvature map; (**b**) corneal thickness, showing greatest thinning inferiorly near the limbus; and (**c**) anterior and (**d**) posterior elevation perpendicular to the thinning.

PMD is characterized by a linear area of corneal thinning located beneath the inferior region of corneal ectasia [10] (Figure 1). The hallmark of PMD is spatial dissociation between the zone of thinning and the ectatic zone, with maximal thinning typically located 1–2 mm from the limbus, while the area of protrusion appears more centrally (Figure 2).

On slit-lamp examination, the area of maximal ectasia, situated above the thinnest portion of the cornea, produces a distinctive “beer belly” appearance (Figure 1) [21]. This hallmark feature, combined with the characteristic “crab-claw” pattern observed on corneal tomography, aids in the clinical diagnosis of PMD. This structural configuration results in a high degree of irregular, against-the-rule astigmatism. However, in many cases, particularly in the early stages, the astigmatism remains relatively regular within the central 3 mm zone, which can contribute to better corrected visual acuity and is relevant when considering contact lens correction. In PMD, the cornea typically remains transparent, without deposits or vascularization, although Vogt’s striae may occasionally be observed [12]. The primary reason patients seek medical attention is a reduction in visual acuity due to irregular astigmatism, often accompanied by ocular discomfort or irritation [10,11,19].

Like other ectatic disorders, PMD is progressive in nature. However, unlike KC, PMD often has a delayed onset and can be more challenging to diagnose, further complicating its management [10].

## 4. Diagnosis

Although videokeratoscopy and corneal tomography have previously been used, corneal tomography—utilizing Scheimpflug imaging and/or a slit-scanning system—remains the gold standard for diagnosing and monitoring all forms of ectatic disease, including PMD. Additionally, anterior segment swept-source optical coherence tomography (AS-SS OCT) can provide complementary diagnostic data by generating detailed pachymetry maps and posterior elevation profiles, further aiding in the assessment and monitoring of PMD [22]. In PMD, the characteristic inferior peripheral thinning, accompanied by subsequent steepening, produces the distinctive “crab-claw” pattern—also referred to as the “kissing birds” (or historically “kissing pigeons”) appearance—on the curvature map of the tomography [13,14,15] (Figure 1). According to Krachmer, in severe cases of PMD, corneal steepness increases significantly, with the thinning area becoming progressively steeper from the centre toward the periphery [19]. This results in a curvature rise of up to 20 diopters.

Following the same principle, elevation maps reveal a heightened tomographical region within the affected area, while pachymetry measurements indicate reduced corneal thickness at the periphery and increased thickness centrally. However, it is crucial to avoid attributing this tomographic pattern exclusively to PMD. Instead, diagnosis should be made in conjunction with the patient’s medical history, slit-lamp examination, and refraction findings [10,23,24].

## 5. Differential Diagnosis

The differential diagnosis for PMD primarily includes other ectatic disorders such as keratoconus (KC) and keratoglobus, as well as peripheral thinning conditions like Terrien’s marginal degeneration. Inflammatory diseases, such as peripheral ulcerative keratitis and Mooren’s ulcer, should also be considered [10].

Early detection of PMD and KC can be challenging, as both conditions exhibit distinct slit-lamp and tomographic/pachymetric features only as they progress. PMD can be readily distinguished from keratoglobus based on its characteristic findings: keratoglobus is congenital and demonstrates diffuse corneal thinning and steepening, whereas PMD typically presents with an inferior “beer-belly” protrusion [16].

Mooren’s ulcer is easily identifiable due to its painful, inflammatory peripheral ulceration with an epithelial defect. Terrien’s marginal degeneration, though rarely confused with PMD, has key distinguishing features—it typically affects younger individuals and progresses slowly, with circumferential thinning often beginning superiorly [25]. Lipid deposition and pseudopterygium formation are also common [26,27]. 

Table 1 outlines the differential diagnoses of PMD.

## 6. Management

Assessment of disease severity and evaluation of the patient’s visual acuity are essential considerations in establishing the appropriate treatment method, either conservative or surgical, for PMD. Given that PMD is a relatively minor constituent of the ectasia spectrum, there is a paucity of research examining the efficacy of treatments for PMD.

## 7. Conservative Management

Early-stage PMD is initially managed with glasses or soft toric contact lenses (SCLs) for regular astigmatism [1,7,11,29], though their efficacy declines with disease progression. Rigid gas permeable (RGP) lenses—including corneal, limbal–miniscleral, and scleral designs—offer superior correction, with studies showing 75.5–95.4% achieving BCVA ≥ 20/40 [28,30]. Bitoric RGP designs enhance stability [31,32,33], while scleral lenses vault over irregular corneas, improving vision in advanced cases (78.7% ≥ 6/9 [34]) despite fitting challenges and tear exchange limitations [35].

Hybrid lenses (e.g., SynergEyes CooperVision, Gilbert, AZ, USA) combine RGP optics with soft lens comfort, achieving 20/30 vision but with 33% complication rates [36]. Newer designs like AirFlex, SwissLens, Prilly, Switzerland, show promise [37], though PMD-specific data remain limited. 

Table 2 summarizes the findings and main takeaway messages from the conservative management options.

## 8. Surgical Management

Surgical management of PMD can be classified into the following types:

(1) Alterations of corneal biomechanics
(2) Toric intraocular lens implantation
(3) Full-thickness surgery
(4) Partial-thickness surgery

### 8.1. Alterations of Corneal Biomechanics

#### 8.1.1. Collagen Cross Linking

Collagen Cross-Linking (CXL) is an established treatment for keratoconus and post-refractive surgery ectasia. The procedure uses UV radiation (365–370 nm) to catalyse photochemical reactions that strengthen the cornea by forming covalent bonds between collagen fibrils [38].

However, PMD presents unique challenges due to its inferior and irregular ectasia, which requires paracentral and peripheral UV application. This can lead to limbal irradiation, potentially compromising limbal stem cells and increasing the risk of limbal stem cell deficiency (LSCD). In PMD, the inferior thinning requires careful adaptation of the CXL protocol. As described by Kymionis et al. [38,39], a decentred UV irradiation zone may help target the ectatic area more effectively. While the use of UV-blocking contact lenses with central apertures to protect the limbal region has been suggested in clinical practice, this technique has not yet been formally evaluated in PMD-specific studies.

A literature review identified 13 studies evaluating CXL in PMD, including 8 case reports. Most employed the standard Dresden protocol, though some used accelerated CXL. The largest study (Iraipour et al., *n* = 40 eyes, 60-month follow-up [40]) found no significant change in mean BCVA post-CXL. While spherical equivalent and steep K-values remained stable in most patients, some experienced progression or improvement. In contrast, Mamoosa et al. [41] reported modest but significant gains in BCVA and reduced keratometry values. Case studies similarly noted improved BCVA and corneal flattening.

Irregular astigmatism in PMD remains difficult to manage, as CXL alone has unpredictable efficacy. A promising alternative is combined PRK and same-day CXL (CXL-plus), which may reduce irregular astigmatism and improve corneal regularity. However, it is important to note that adding PRK carries a higher long-term risk of ectasia progression compared to CXL alone.

Kymionis et al. (2009) first documented CXL in PMD, reporting stable ectasia and improved BCVA at 12 months [38,39]. The same group later observed similar success in a PMD patient with prior intracorneal ring segments (ICRS) [42].

Another study (*n* = 8 eyes) evaluated CXL-plus (transepithelial PTK + CXL) in PMD, showing improved UDVA (1.05 ± 0.33 to 0.41 ± 0.27 logMAR) and reduced astigmatism (−6.83 ± 2.33 D to −4.71 ± 1.89 D) at one year [43]

Cagil et al. used simultaneous transepithelial PTK + accelerated CXL, achieving reduced cylinder and spherical equivalent over 36 months—though without BCVA improvement [44]. 

Table 3 summarizes the key surgical interventions for PMD, comparing their mechanisms, indications, benefits, and limitations.

#### 8.1.2. Intrastromal Corneal Rings

Intracorneal ring segments (ICRS) are medical devices composed of polymethyl methacrylate (PMMA) that are surgically implanted into the corneal stroma to modify its curvature and improve optical performance. The corneal incision for ICRS insertion can be created either manually using pocket micro-dissectors or with a femtosecond laser, typically at 50% corneal depth.

Several studies have investigated ICRS outcomes in PMD. ICRS have been shown to improve visual acuity and corneal topography in early to moderate pellucid marginal degeneration (PMD), reducing astigmatism and enhancing best-corrected visual acuity (BCVA) [45]. However, long-term complications—including regression, extrusion, and stromal melting—remain a concern. Recently, biologic alternatives such as allogenic corneal ring segments (CAIRS) have been introduced for keratoconus and may offer a promising future option for PMD [46]. These options are discussed in more detail in the Unmet Needs and Future Directions section.

The largest study by Hashemian et al. [45] utilized a femtosecond laser to implant single-segment Intacs SK rings (Addition Technology, USA) in 36 eyes of 26 patients. After six months, patients demonstrated a 2 D reduction in astigmatism, a 1.5 D decrease in mean keratometry, and improved BCVA from logMAR 0.42 to 0.16. Similarly, Kubaloglu et al. [47] reported outcomes using Keraring segments (Mediphacos, Brazil) with femtosecond laser-assisted implantation. At a mean follow-up of 30.7 months, astigmatism decreased by 2 D, maximum keratometry (Kmax) reduced by nearly 4 D, spherical equivalent improved by 3 D, and BCVA improved from logMAR 0.88 to 0.35. These findings were corroborated by Mularoni et al. [48], who observed comparable reductions in astigmatism and visual acuity gains.

Despite demonstrating efficacy in early-to-moderate PMD [10,45,49], ICRS use remains limited due to several challenges, including postoperative regression, unpredictable long-term visual outcomes, and the risk of sight-threatening complications such as corneal melting.

### 8.2. Toric Intraocular Lens Implantation

As with other surgical interventions for PMD, current evidence regarding toric intraocular lenses (IOLs) remains limited, underscoring the need for larger prospective studies to establish their safety and efficacy. Nevertheless, existing research—whether utilizing phakic or pseudophakic toric IOLs—demonstrates promising outcomes.

Balestrazzi et al. [50] reported improved BCVA and significant reductions in both sphere and astigmatism in 11 PMD eyes undergoing cataract surgery with pseudophakic IOLs, with no postoperative visual disturbances. Similar outcomes were observed by Matalia et al. [51], Bahar et al. [52], Han et al. [53], and Luck et al. [54], all noting enhanced BCVA and refractive stability following toric IOL implantation during cataract surgery.

For phakic toric IOLs, Camoriano et al. [55] (*n* = 10) documented a BCVA improvement from 20/20 to 20/18 and a reduction in spherical equivalent from −6.71 ± 0.9 D to −0.58 ± 0.1 D, while de Vries et al. [56] (*n* = 1) also reported satisfactory visual and refractive outcomes. Notably, one patient in Camoriano’s study required IOL explantation due to haloes and glare.

While toric IOLs demonstrate promising visual outcomes in PMD, several important limitations must be considered. First, the characteristic corneal ectasia in PMD often compromises biometry reliability, significantly complicating IOL power calculation and selection [57]. More fundamentally, while toric IOLs effectively address refractive errors, they do not correct the underlying corneal ectasia or provide biomechanical stabilization. This critical limitation means the procedure fails to halt disease progression, potentially allowing continued corneal deterioration that may require optical correction (e.g., spectacles) or additional surgical interventions in the future [51].

### 8.3. Full-Thickness Surgery

#### 8.3.1. Penetrating Keratoplasty

##### Penetrating Keratoplasty (PK) in PMD

Penetrating keratoplasty (PK) remains a fundamental surgical intervention for advanced PMD cases, offering complete replacement of the irregular corneal tissue. The procedure presents unique challenges in PMD due to the characteristic inferior paracentral thinning, requiring large eccentric grafts that complicate both surgical execution and postoperative visual rehabilitation. Achieving optimal donor tissue centration proves particularly difficult, often resulting in challenging wound closure and significant postoperative astigmatism. Studies by Cherry et al. [58] and Tuberville et al. [59] have demonstrated that these peripherally placed grafts carry increased risks of corneal neovascularization, glaucoma, and graft rejection when positioned near the limbal vasculature.

The most comprehensive longitudinal study of PK in PMD by Tzelikis et al. [60] followed patients for nine years, documenting visual acuity improvement from 20/153 preoperatively to 20/43 at one year postoperatively. While other investigations reported acceptable postoperative astigmatism ranging from 0.75 to 2.46 diopters, they simultaneously noted concerning graft rejection rates approaching 50%. Given that PMD patients typically maintain healthy endothelial cell function, most surgeons reserve PK for cases refractory to other treatment modalities [61,62]. Even with successful graft healing, residual astigmatism frequently persists and can substantially compromise visual acuity. Consequently, many patients require supplemental optical correction—typically with rigid gas-permeable (RGP) or scleral contact lenses—to attain functional vision. This challenge is particularly relevant in PMD, where corneal irregularity often persists despite tectonic stabilization.

#### 8.3.2. PK Surgical Alternatives

##### Full-Thickness Crescentic Wedge Resection

First described by Durand et al. in 1991 [62,63], full-thickness crescentic wedge resection eliminates the need for donor tissue by excising the thinned corneal stroma and approximating the remaining tissue. While offering faster recovery times compared to PK, this technique presents challenges with postoperative astigmatic drift, as documented in MacLean’s case series of 10 eyes [64]. Busin et al. [65] attempted to address this limitation by combining wedge resection with corneal relaxing incisions, achieving more stable refractive outcomes in their 10-eye series.

### 8.4. Partial-Thickness Procedures

Partial-thickness variations of crescentic wedge resection maintain endothelial integrity while still addressing the corneal ectasia. Genc et al. [66] reported BCVA improvement from 20/125 to 20/32 in their 10-eye series, though they observed a 10% rate of intraoperative microperforation. Technical refinements including pneumatically-assisted and femtosecond laser-assisted techniques developed by Tsatsos et al. [67,68] have enhanced the precision and safety of these procedures. Kymionis et al. [69] further advanced the technique by combining sectoral lamellar resection with corneal cross-linking, demonstrating stable visual outcomes at 14-month follow-up without disease progression.

#### 8.4.1. Crescentic Lamellar Keratoplasty (CLK)

CLK represents a tissue-sparing alternative that replaces only the diseased anterior stroma while preserving the patient’s endothelium. This approach theoretically reduces rejection risk compared to PK but introduces new challenges, including graft–host interface opacification and significant postoperative astigmatism. The procedure demands considerable surgical expertise and currently lacks robust long-term outcome data from large prospective studies [70,71].

#### 8.4.2. Deep Anterior Lamellar Keratoplasty (DALK)

DALK has emerged as a particularly promising alternative to PK for PMD, combining the advantages of lamellar surgery with excellent visual outcomes. The largest DALK series in PMD by Al Torbak et al. [72] demonstrated visual improvement from 0.9 to 0.4 LogMAR with a reduction in astigmatism from −8 to −4.3 diopters. The procedure carries technical challenges, with a 50% success rate for baring Descemet’s membrane and a 12.5% perforation rate in their 16-eye series. Smaller case reports by Kodavoor [73] and Millar [74] have corroborated these positive outcomes while emphasizing the technique’s steep learning curve.

#### 8.4.3. Historical Techniques

Older surgical approaches, including lamellar thermokeratoplasty and epikeratoplasty, have largely been abandoned due to their association with high complication rates and unpredictable outcomes. These techniques have been replaced by the more refined lamellar procedures discussed above [74].

The surgical management of PMD continues to evolve, with current evidence suggesting that lamellar techniques may offer distinct advantages over traditional penetrating keratoplasty in appropriate cases. The relative rarity of PMD has limited the availability of large comparative studies, necessitating careful individualization of surgical approach based on patient-specific anatomical considerations and surgeon expertise. Future research should prioritize standardized outcome reporting and extended follow-up to better establish optimal treatment algorithms for this challenging corneal disorder. 

Table 4 summarizes the benefits and limitations of full-thickness and partial-thickness surgical procedures for PMD.

## 9. Conclusions

### Pellucid Marginal Degeneration: Diagnostic and Therapeutic Challenges

Pellucid marginal degeneration (PMD) represents a complex corneal ectatic disorder characterized by progressive inferior peripheral thinning and significant irregular astigmatism, leading to substantial visual impairment. While relatively rare, PMD presents distinct diagnostic and management challenges that often necessitate a coordinated, multidisciplinary approach to patient care.

**Diagnostic Considerations and Early Intervention:** Timely diagnosis remains critical in PMD management, with Scheimpflug-based corneal tomography emerging as the imaging modality of choice for detecting characteristic topographic patterns. This advanced imaging allows for early identification of the disease’s hallmark inferior “beer-belly” protrusion and peripheral thinning, enabling prompt intervention before significant visual deterioration occurs.

**Therapeutic Approaches:** The treatment paradigm for PMD follows a progressive approach:

**Conservative Management:** In mild to moderate cases, rigid gas permeable (RGP) or scleral contact lenses serve as first-line therapy, often providing excellent visual rehabilitation by neutralizing irregular astigmatism. These modalities frequently restore functional vision and significantly improve patients’ quality of life without surgical intervention.

**Surgical Options:** For progressive cases, collagen cross-linking (CXL) has shown promise in stabilizing corneal ectasia, while intracorneal ring segments (ICRS) can help regularize corneal topography. In patients with concurrent cataract or stable ectasia, toric intraocular lenses (IOLs) may offer effective refractive correction. However, these approaches require careful patient selection and thorough preoperative evaluation.

In advanced disease, penetrating keratoplasty (PK) or deep anterior lamellar keratoplasty (DALK) remain the definitive surgical options. While effective, these procedures carry inherent risks including graft rejection (particularly with PK), persistent astigmatism, and extended visual rehabilitation periods. Recent evidence suggests DALK may offer advantages in PMD cases due to its endothelial-sparing nature and lower rejection rates.

**Unmet Needs and Future Directions:** Despite these available treatment modalities, several critical limitations persist in PMD management. The field lacks standardized treatment algorithms, robust long-term outcome data for various interventions, and targeted therapies addressing the underlying disease mechanisms. These knowledge gaps highlight the need for future research focusing on multicenter studies to establish evidence-based protocols, basic science investigations to uncover novel therapeutic targets, and technological advancements in corneal imaging analytics. Additionally, refinement of lamellar surgical techniques and development of customized intracorneal ring segment designs may further improve outcomes for this challenging condition. A promising surgical approach for PMD is the use of Corneal Allogenic Intrastromal Ring Segments (CAIRS), which replaces synthetic PMMA rings with semicircular donor stromal tissue. In keratoconus, CAIRS has demonstrated improved biocompatibility, fewer complications (e.g., extrusion or stromal melting), and encouraging visual outcomes. Jacob et al. reported early safety and efficacy in keratoconus patients, with no implant-related adverse events [45]. Although CAIRS has not yet been extensively studied in PMD, its potential for better biologic integration and lower risk of foreign body rejection makes it a compelling alternative—particularly for high-risk eyes that may not tolerate synthetic implants. Future studies evaluating PMD-specific outcomes are needed to confirm its long-term efficacy.

Ultimately, optimal management of PMD requires careful staging and individualized treatment approaches guided by the specific characteristics and progression pattern in each patient. While current therapeutic options can effectively address various disease manifestations, significant opportunities remain for improving both diagnostic precision and treatment outcomes. A concerted research effort focusing on long-term outcomes, surgical innovation, and pathophysiological understanding will be essential to advance the care of patients affected by this vision-threatening disorder. Through such advancements, clinicians may one day be better equipped to preserve and restore vision in individuals with PMD, ultimately improving their quality of life and visual potential.

## Figures and Tables

**Table 1:** Differential diagnoses of PMD.

Disease	Key Characteristics	Differentiating Features
Pellucid Marginal Degeneration (PMD)	- Inferior peripheral corneal thinning (crescent-shaped). - Νon-inflammatory. - Irregular astigmatism. - “Beer-belly” appearance on tomography.	- Thinning is localized to the inferior periphery. - No lipid deposits or vascularization. - Crab-claw pattern on tomography.
Keratoconus (KC)	- Central or paracentral corneal thinning. - Cone-shaped protrusion. - Irregular astigmatism.	- Thinning and protrusion are central or paracentral. - No inferior crescent-shaped thinning. - Different tomography pattern (not usually crab-claw).
Keratoglobus	- Congenital, diffuse corneal thinning. - Globular protrusion of the entire cornea.	- Thinning and protrusion are global, not localized. - Onset is congenital, unlike PMD.
Terrien’s Marginal Degeneration	- Peripheral circumferential thinning. - Lipid deposits and pseudopterygium. - Non-inflammatory.	- Thinning is circumferential, not inferior. - Lipid deposits and pseudopterygium are common. - No crab-claw tomography.
Peripheral Ulcerative Keratitis (PUK)	- Inflammatory peripheral corneal thinning. - Associated with systemic autoimmune diseases.	- Painful, inflammatory, and often associated with systemic disease. - Epithelial defect and vascularization.
Mooren’s Ulcer	- Painful, inflammatory peripheral ulcer. - Epithelial defect. - No systemic association.	- Severe pain and inflammation. - Epithelial defect present. - No lipid deposits or pseudopterygium.
Acute Hydrops	- Fluid accumulation in the cornea due to Descemet’s membrane rupture. - Associated with advanced ectatic diseases [28].	- More common in keratoconus but higher risk in PMD. - Sudden onset of corneal edema and vision loss.

**Table 2:** Summary of the findings and main takeaway messages from the conservative management options.

Treatment Modality	Indications	Benefits	Limitations
Glasses	Early stages of PMD with mild refractive errors	- Simple and non-invasive - First-line option for mild astigmatism	- Limited effectiveness in advanced stages - Cannot correct irregular astigmatism
Soft Contact Lenses	Early stages of PMD with regular astigmatism	- Easy to use and comfortable - Effective for regular astigmatism	- Less effective as irregular astigmatism progresses - Limited in advanced PMD
Corneal Rigid Gas Permeable (RGP) Lenses	Moderate to advanced PMD with irregular astigmatism	- Provide better visual acuity in irregular astigmatism - Improved stability	- Fitting can be challenging - Require gradual adjustment of lens diameter
Limbal/Mini-Scleral RGP Lenses	Advanced PMD with significant corneal irregularity	- Better centration and stability compared to corneal RGP lenses	- Require expertise in fitting - May still dislodge in severe cases
Scleral RGP Lenses	Advanced PMD with inferior corneal protrusion and severe irregularity	- Excellent stability and centration - Vault over the cornea, reducing irregularity impact	- Limited availability of trained practitioners - Reduced tear turnover and debris buildup
Hybrid Contact Lenses	Patient intolerance to RGP lenses or needing combined comfort and visual acuity	- Combines RGP vision correction with soft lens comfort	- Prone to tearing at the RGP-soft lens junction - Risk of oxygen permeability issues
Third-Generation Hybrid Lenses	Patients with sensitivity to RGP lenses	- Improved visual acuity (up to 20/30) - Better comfort for sensitive patients	- Higher complication rate (e.g., conjunctivitis, edema, allergic reactions)
Emerging Hybrid Lenses (e.g., AirFlex)	Emerging option for PMD management	- Promising early results - Combines comfort and visual correction	- Limited long-term data and research

**Table 3:** Summary of key surgical interventions in PMD: mechanisms, benefits, and limitations.

Procedure	Mechanism	Indications	Key Benefits	Limitations/Risks
Collagen Cross-Linking (CXL)	UV-A-induced photochemical bonding of collagen fibrils to increase corneal rigidity	Progressive PMD with sufficient corneal thickness	- May halt progression of ectasia - Improved corneal biomechanics - Minimally invasive	- Variable visual outcomes - Limited efficacy in highly irregular astigmatism - Risk to limbal stem cells in inferiorly thinned areas
CXL-Plus (PRK + CXL)	Surface ablation followed by CXL for refractive and structural improvement	Select PMD cases with central astigmatism and adequate stromal thickness	- May reduce irregular astigmatism - Potential for better uncorrected visual acuity	- Higher biomechanical risk, especially in peripherally thinned corneas - Risk of ectasia progression if poorly selected
Intrastromal Corneal Ring Segments (ICRS)	Synthetic PMMA ring segments reshaping the corneal curvature	Mild to moderate PMD with contact lens intolerance	- Reduces astigmatism (2–4 D) - Improves BCVA - Reversible and minimally invasive	- Unpredictable long-term outcomes - Possible complications (e.g., extrusion, infection) - Limited effect in advanced PMD
Toric Intraocular Lenses (IOLs)	Astigmatism correction via toric optics during cataract or refractive lens surgery	PMD with coexisting cataract or stable ectasia	- Reduces refractive astigmatism - Good BCVA in selected cases - Single-step solution in cataract surgery	- Does not treat underlying ectasia - Risk of misalignment - IOL power calculation can be inaccurate in irregular corneas

**Table 4:** Summarizes the benefits and limitations of full-thickness and partial-thickness surgical procedures for PMD.

Procedure	Benefits	Negatives (Limitations)
1. Penetrating Keratoplasty	- Removes all irregular corneal tissue, providing a clear optical surface. - Improves visual acuity significantly (e.g., 20/153 to 20/43 in Tzelikis et al. [60]).	- High risk of graft rejection (up to 50%). - Postoperative astigmatism is common and challenging to manage. - Requires large, eccentric grafts, increasing surgical complexity. - Risk of complications like neovascularization, glaucoma, and infection.
2. Full-Thickness Crescentic Wedge Resection	- Does not require donor tissue. - Shorter recovery time compared to PK. - Reduces astigmatism in some cases.	- Limited evidence and small sample sizes in studies. - Risk of astigmatic drift. - Intraoperative challenges and potential for complications like microperforation.
3. Partial-Thickness Crescentic Wedge Resection	- Preserves endothelial layer, reducing rejection risk. - Improves BCVA (e.g., 20/125 to 20/32 in Genc et al. [65]). - No donor tissue required.	- Risk of intraoperative microperforation (14% in some studies). - Suture-related complications (e.g., infiltration in 34% of cases). - Limited long-term data.
4. Crescentic Lamellar Keratoplasty	- Preserves endothelium, reducing rejection risk. - Improves visual outcomes with contact lenses or spectacles. - Maintains structural integrity of the eye.	- Postoperative astigmatism is common. - Risk of graft–host interface opacification. - Technically challenging with a steep learning curve.
5. Deep Anterior Lamellar Keratoplasty	- Preserves recipient endothelium, reducing rejection risk. - Lower postoperative astigmatism compared to PK. - Shorter steroid therapy duration and fewer complications.	- Technically demanding and time-consuming. - High risk of intraoperative perforation, especially in corneas with hydrops. - Limited improvement in BCVA compared to PK.
6. Lamellar Thermokeratoplasty and Epikeratoplasty	- Historical procedures with limited use today.	- High complication rates. - Outdated and replaced by safer, more effective techniques.
